# Multifunctional Nanomaterials and Their Applications in Drug Delivery and Cancer Therapy

**DOI:** 10.3390/nano5041690

**Published:** 2015-10-14

**Authors:** Mathangi Srinivasan, Mehdi Rajabi, Shaker A. Mousa

**Affiliations:** The Pharmaceutical Research Institute, 1 Discovery Drive, Rensselaer, NY 12144, USA; E-Mails: mathangi.srinivasan@acphs.edu (M.S.); mehdi.rajabi@acphs.edu (M.R.)

**Keywords:** cancer therapy, drug delivery, multifunctional nanoparticles, nanotechnology, tumor targeting, imaging

## Abstract

The field of nanotechnology has led to the development of many innovative strategies for effective detection and treatment of cancer, overcoming limitations associated with conventional cancer diagnosis and therapy. Multifunctional nanoparticle systems can integrate imaging, targeting and treatment moieties on the surface and in the core, resulting in targeted delivery of the imaging or treatment modalities, specifically to the tumor. Multifunctional nanoparticles also enable simultaneous delivery of multiple treatment agents, resulting in effective combinatorial therapeutic regimens against cancer. In this review, various multifunctional nanoparticle systems that feature a variety of targeting moieties for *in vitro* and/or *in vivo* cancer imaging and therapy are discussed.

## 1. Introduction

Multifunctional nanoparticle-based platforms of anti-cancer drug delivery have paved the way for innovative therapies that are more efficacious, less invasive and less toxic. Nanoparticle carriers provide significant advantages over conventional therapy modalities [1]. Nanoparticles (NPs) have higher water solubility; therefore, they can be used as a carrier of insoluble drugs, eliminating the need for toxic organic solvents and their adverse side effects. Nanocarriers can be designed to tailor the release kinetics using environmental (pH) or external stimuli (ultrasound, heat). This advantage of controlled release prevents premature dissociation of the drug from the nanoshell before it is at the tumor site, minimizing drug accumulation in other healthy tissues and organs, and therefore, decreasing the systemic toxicity associated with the drug.

A significant advantage of using nanosized carriers is their ability to be targeted actively to the tumor sites. Conventional chemotherapy drugs are cytotoxic and kill actively dividing cancer cells, but can also affect other healthy dividing cells. The surface area/volume ratio and the chemistry of NPs afford the attachment of cancer-specific molecules on the NP surface that can specifically bind to their targets on the cancer cell. Internalization of the NP into the cell results in higher cellular uptake of the drug/active agent and higher anti-tumor activity. Targeted delivery isolates the effect of the drug to just the cancer cells expressing the targeted molecule, decreasing the systemic toxicity and the side effects of the drug or active agent, some of which could be life threatening [2].

**Figure 1:**
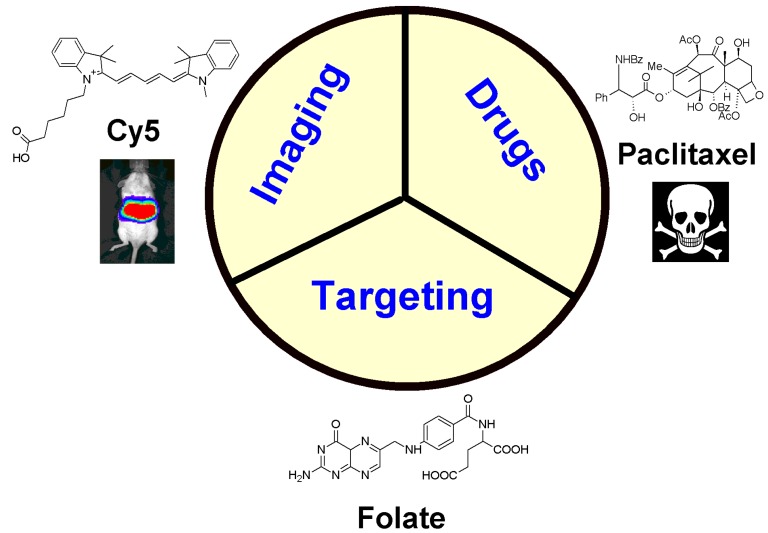
The three main classes of functionalities that comprise a multifunctional nanoparticle for cancer diagnostics and therapy. A tumor-specific targeting moiety, such as the folate molecule, recognizes the folate receptor on the tumor cell surface to provide directed delivery of an imaging probe, for example the Cy5 fluorescent molecule, and/or the treatment agent, for example the chemotherapy drug paclitaxel.

In contrast to monofunctional NPs that deliver only a single payload of drug or active agent, multifunctional NPs can integrate various functionalities inside the core of the NP or on the NP surface to synergistically achieve maximal anti-tumoral activity (Figure 1). It has become increasingly evident that successful therapeutic regimens involve more than one drug and its target [3,4]. Current clinical trials are already testing various combinations of treatment options to achieve the best results [5,6]. In cancer therapy, multifunctional NPs are being researched in the delivery of therapeutic agents that include small molecule drugs, antigenic proteins, aptamer sequences and molecular components (DNA, siRNA, shRNA and miRNA) [7].

Multifunctional NPs can also be loaded with imaging agents or molecules to provide diagnostic information during optical imaging, magnetic resonance and photothermal detection [8]. Overall, they can be engineered to detect cancer cells, deliver treatment agents and monitor treatment response, thus integrating diagnosis and treatment in real time. In this review, we discuss the various types of materials used to synthesize multifunctional NPs for cancer imaging and therapy and summarize recent and ongoing research in the fabrication of these designer NPs against cancer. We highlight the three main components that make up a multifunctional NP in cancer drug delivery and imaging: the targeting ligand, the anti-cancer therapeutic agent and the imaging modality.

## 2. Nanomaterials Used in the Synthesis of Multifunctional Nanoparticles

**Figure 2:**
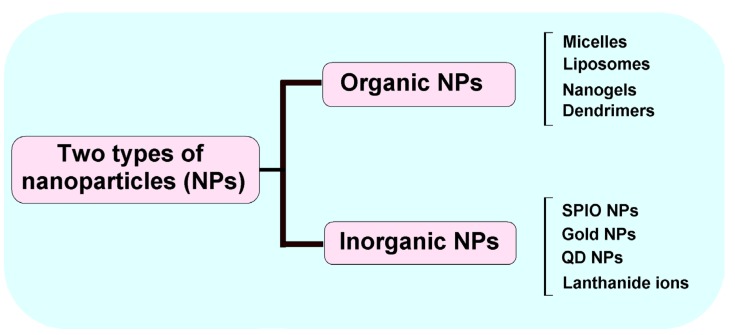
Inorganic and organic nanoparticle (NP) materials. Multifunctional NPs can be synthesized using two basic types of NPs; organic (micelles, liposomes, nanogels and dendrimers) and inorganic (superparamagnetic iron oxide (SPIO), gold, quantum dots (QD) and lanthanide ions.

A number of organic and inorganic materials have been used to fabricate multifunctional NPs with their own distinctive architecture and attached functionalities (Figure 2), and they have been evaluated for effective drug delivery to tumors [9].

**Figure 3:**
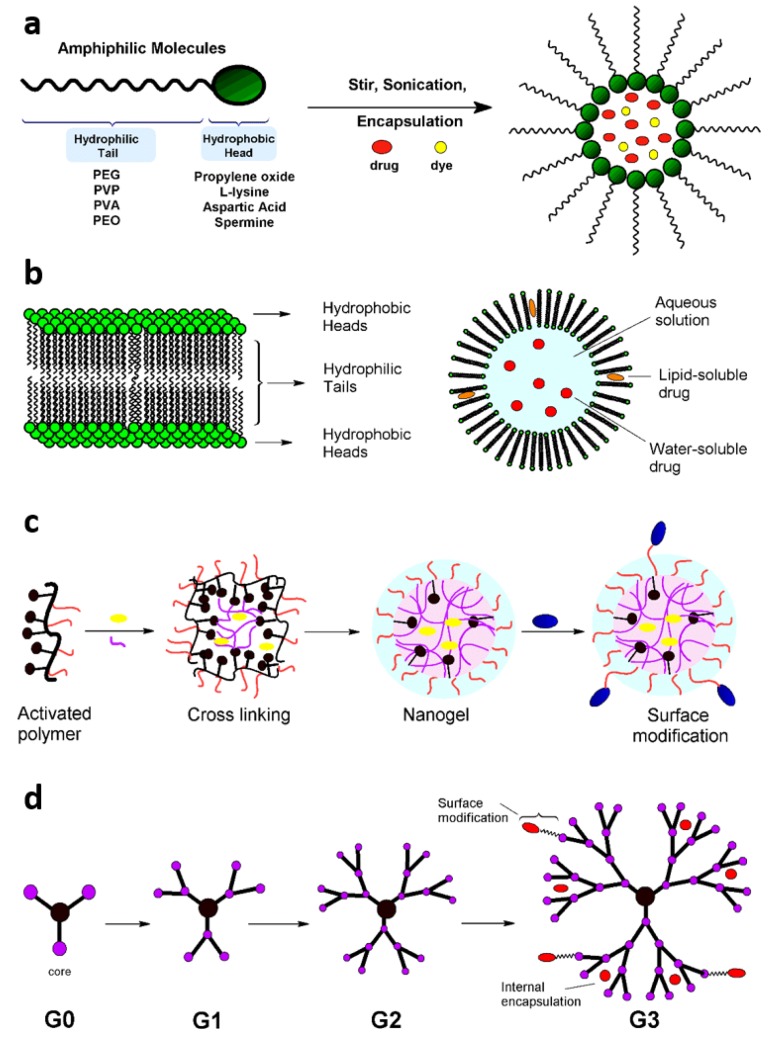
Organic nanoparticles (NPs): nanomicelles, liposomes, nanogels and dendrimers. (**a**) Nanomicelles are constructed by the assembly of amphiphilic blocks with a hydrophobic head and a hydrophilic outer tail. Imaging and treatment agents are encapsulated inside the hydrophobic core; (**b**) Liposome NPs are made up of lipid bilayers that carry drug inside the hydrophobic core or inside the lipid bilayer; (**c**) Polymeric nanogels are synthesized by crosslinking activated polymer chains, creating an inner porous space that can accommodate multiple payloads and imaging moieties; (**d**) Dendrimers are highly branched polymers that are synthesized by repeated branching cycles. The inner core and the outer branches provide multiple points of conjugation of imaging, targeting and treatment modalities.

Organic or polymeric NPs, such as micelles, liposomes, nanogels and dendrimers, are attractive building blocks for multifunctional NPs due to their versatile surface and core chemistry, high degree of biodegradability, effective endocytosis by the target cell and high payload loading efficiency [7]. Nanomicelles are constructed using amphiphilic blocks with the core made up of hydrophobic blocks, such as propylene oxide, l-lysine, aspartic acid, d,l-lactic acid and spermine, while the outer corona consists of hydrophilic blocks, such as poly(ethylene glycol) (PEG), poly(*N*-vinyl-2-pyrrolidone) (PVP) and poly(vinyl alcohol) [10]. Micellar NPs can encapsulate hydrophobic drugs and imaging agents in their core, thus circumventing the need for toxic organic solvents (Figure 3a). Liposomes are constructed from bilayers of enclosed spherical structures, such as phospholipids, cholesterol and other similar lipid molecules. Liposomes are ideal for the encapsulation in their hydrophilic core of nucleic acid-based components, such as siRNA and plasmid DNA, to be targeted to tumor sites (Figure 3b) [11]. Polymeric nanogels are fabricated by cross-linking polymer chains to create an inner porous space that can accommodate a large volume of payload, and therefore, are conducive for simultaneous delivery of multiple treatment modalities (Figure 3c) [12]. Dendrimers are yet another group of organic polymeric NPs that can accommodate multiple functionalities at their terminal groups. Dendritic molecules, such as polyamidoamine (PAMAM), poly(propylene imine) polyamide, polyglycerol (PG) and triazine, are synthesized by assembling monomeric subunits to form uniform tree-like structures that allow for encapsulation of the drug payload in the internal star structure, while the branches can be complexed with functionalities, such as targeting ligands, imaging agents and other drugs (Figure 3d) [13].

**Figure 4:**
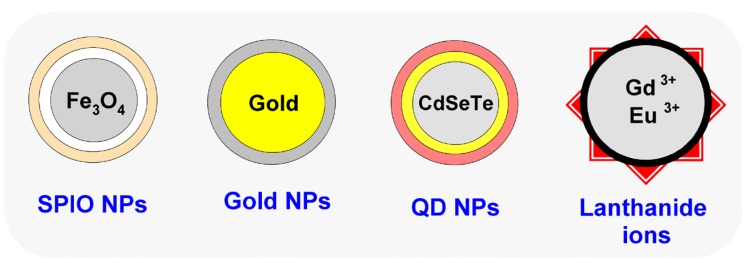
Inorganic NPs. Four important inorganic nanomaterials used in the construction of multifunctional NPs are SPIO, gold, QD and lanthanide ions.

Inorganic NPs exhibit photochemical, photothermal and magnetic properties that lend themselves to detection and diagnostic applications (Figure 4). Additionally, their rigid outer surface and the durable core can be complexed with a variety of drug/active agent payloads. Magnetic NPs, such as superparamagnetic iron oxide particles, have been used in image contrast and magnetic resonance to detect the presence of tumors. Modifications to the surface of these molecules, such as coating with surfactants and bioconjugation between the carboxyl and amine groups, make them amenable to complexation with targeting functionalities that can enhance the diagnostic capability of these NPs [14]. Gold NPs have optical and electric properties that can be exploited in tumor imaging [15]. Quantum dots are semiconductor NPs that have narrow emission and a broad range of absorption bands that have been predominantly used in fabricating imaging probes [16]. Gold NPs and quantum dots have been fabricated along with the organic polymers to form hybrid systems that can integrate packaging of the drug/active agent and diagnostic capabilities for effective drug delivery accompanied by real-time monitoring of treatment response and tumor activity [17,18]. Lanthanide-doped NPs are a class of fluorescent NPs that allow for conversion of low energy light, such as near infra-red (NIR), to high energy visible light or UV emission. This upconversion effect that takes place by multiphoton absorptions or energy transfer processes along with the long-lasting luminescence of the lanthanides has been exploited in various cancer diagnostic trials [19,20], as well as in treatment modalities, such as imaging-guided NIR laser irradiation of tumors [21].

## 3. Multifunctional Nanoparticles Used in Cancer Therapy

The nano-encapsulation of traditional chemotherapy drugs, such as doxorubicin (DOX), paclitaxel (PXL), docetaxel, cis-diamminedichloroplatinum (II) (cisplatin), cytarabine, vincristine (VCR), vinblastine (VLB), vinorelbine (microtubule inhibitor), camptothecin, lurtotecan and irinotecan, opened an impactful avenue in cancer treatment modalities. Doxil^®^, a long-acting PEGylated liposomal formulation of doxorubicin, has been through successful preclinical and clinical testing for the treatment of ovarian cancer [22]. Currently, PEGylated liposomal doxorubicin is in use in phase 2/phase 3 clinical trials for stage 1–3 triple negative breast cancer [23], diffuse large B-cell lymphoma [24], refractory multiple myeloma [25] and melanoma [26]. Phase 3 trials of aggressive metastatic breast cancer treated with Abraxane^®^ or nab-paclitaxel (nano-albumin-encapsulated paclitaxel) resulted in a longer median time to progression, a higher overall response rate and a longer median overall survival in patients compared to solvent-based paclitaxel. A number of clinical trials have now been initiated using these nanodrugs in combination with other biologic and/or cytotoxic agents to treat early and metastatic breast cancer [5,27], advanced non-small lung cancer in patients who cannot be treated by radiation or surgery [28] and advanced pancreatic cancer [29]. Multifunctional NP platforms offer the feasibility to engineer these novel drug combinations in a single drug delivery system for maximal therapeutic efficiency.

A comprehensive understanding of the chemistry and the pharmacokinetics of these various drugs and other biologic agents that have been utilized in the treatment of multiple cancers is imperative for the construction of sophisticated multifunctional drug platforms. The combination of hydrophilic (e.g., doxorubicin) or hydrophobic (e.g., paclitaxel) drugs with negatively-charged siRNA or DNA can target different metabolic pathways of tumor cells, resulting in a multipronged attack on the cells [30]. In a breast cancer model, Wang *et al*. used an amphiphilic triblock copolymer, poly(*N*-methyldietheneamine sebacate)-co-[(cholesteryl oxocarbonylamido ethyl) methyl bis(ethylene)ammonium bromide] sebacate, or P(MDS-co-CES, to co-deliver paclitaxel and plasmid DNA encoding IL-12 or an siRNA against Bcl-2 [31]. Both of these cationic core shell NPs suppressed cancer growth more efficiently than individual delivery of the two components. Zhang *et al*. used a temperature-sensitive pentablock copolymer along with a pluronic polymer to simultaneously deliver plasmid DNA and paclitaxel to the name of a human ovarian cancer cell line (SKOV3) ovarian carcinoma cells and demonstrated the synergistic and sustained delivery of both agents [32]. Similarly, nanostructured calcium carbonate was used to deliver doxorubicin hydrochloride and p53 expression plasmids, yielding enhanced cell apoptosis in cervical carcinoma HeLa cells [33].

Several nanoformulations of siRNA and shRNA inhibitory sequences against genes that suppress the expression of the various functional and metabolic pathways of cancer cells have been developed and successfully used to inhibit the growth and progression of cancer. Biswas *et al*. developed a poly(ethylene glycol)-dioleoylphosphatidyl ethanolamine (PEG-DOPE)-modified G(4)-PAMAM nanocarrier that could deliver a drug-siRNA combination payload to tumor cells. The lipid modification of cationic polymers increased the transfection efficiency, and the micellar dendrimer system yielded higher stability and protection of the siRNA against enzymatic degradation [34]. Paclitaxel combined with siRNA complexes against VEGF and PlK-1 was used in studies using multifunctional cationic micelles [35,36], resulting in increased endocytosis in tumor cells and higher tumor suppression. Drug payloads, such as paclitaxel or doxorubicin, complexed with siRNA against multi-drug transporter genes, such as the P-glycoproteins and the adenosine triphosphate (ATP) binding cassette (ABC) transporters overexpressed in cancer cells, inhibit gene expression of these proteins, resulting in increased drug retention and higher cytotoxicity [37].

The next section discusses the other arm of an efficient anti-cancer multifunctional NP: the targeting moiety that can specifically recognize tumor cells and maximally deliver drug/active agent payloads to the tumor site.

## 4. Tumor-Specific Targeting

One of the major advantages of nanotherapy in cancer is its capability to distinguish between cancerous and healthy tissue and, therefore, to minimize the systemic toxicity associated with chemotherapy. The size and pharmacokinetic properties of the NPs ensure that they accumulate at tumor sites. This is done through passive targeting, which is the preferential accumulation of NPs at tumor sites and is dependent on the pathophysiology of the tumor mass as a whole and the uniqueness of the tumor microenvironment. The irregular and porous blood vessel network surrounding the tumor allows the penetration of NP drug delivery complexes in a size-dependent manner. Passive delivery can be further enhanced by modification of the nanomaterials’ surface using different hydrophilic spacers, such as poloxamer, polyethylene oxide (PEO), PEG, lauryl ethers and polysorbate [38].

**Figure 5:**
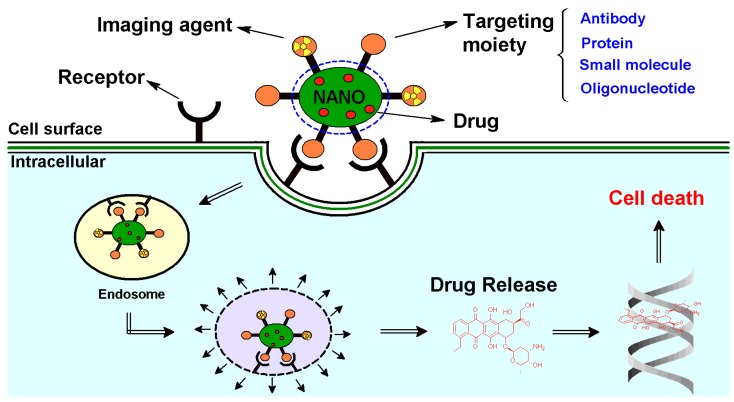
Schematic diagram representing the mode of action of targeted multifunctional nanoparticle (NP). The NP encapsulating a chemotherapy drug is targeted to the cancer cell surface by the cancer cell-specific ligand. The NP then binds to the cancer cell surface by recognizing the receptor, resulting in internalization of the NP by endocytosis. Inside the cancer cell, the NP undergoes endosomal escape, leading to the release of the treatment cytotoxic drug, which then activates consecutive steps, resulting in cell death.

Active targeting is a more molecularly-driven process. It involves the attachment of affinity ligands on the surface of an NP that identify uniquely overexpressed molecules on the tumor cell surface to promote higher and more sustained drug interactions with its pharmacological target. The ligands are attached to the nanosurface by various chemical surface modifications [39]. Antibodies (anti-Her2, anti-CD44, anti-CD24), proteins (transferrins), small bioactive molecules (biotin, folate, galactose, mannose and glucose), peptides (like l-arginine glycine l-aspartic acid (RGD)) and oligonucleotides (aptamers) have been used as ligands to recognize target receptors on the tumor cells (Figure 5) [2].

Anti-HER2 targeting antibodies conjugated to liposome-grafted PEG chains strongly increased the uptake of the NPs in HER2-expressing breast tumors. Liposomes without the anti-HER2 antibody were concentrated in the perivascular and stromal spaces and were internalized by the cancer-associated macrophages, reducing the availability to the tumors [40]. The transferrin receptor, which is overexpressed on cancer cell surfaces, has been targeted using whole transferrin proteins conjugated to NPs [41]. In a prostate cancer preclinical model, transferrin-conjugated PLGA NPs with encapsulated paclitaxel displayed better anti-tumor activity than either free paclitaxel or NPs with paclitaxel that did not have the transferrin conjugation. Smaller peptide sequences have also been used as targeting moieties; the smaller molecular sizes of these shorter peptide chains afford higher stability and easier synthesis and conjugation to nanosurfaces. In an ovarian carcinoma model, a luteinizing hormone-releasing hormone (LHRH) peptide has been used to target cells that overexpress the receptor for this hormone. Nano-polymers, such as dendrimers, micelles and liposomes, that were intercalated to doxorubicin and siRNA were conjugated with targeting peptides toward CD44 or multidrug resistance protein-1 (MRP1) and displayed the highest tumor growth suppression in mice that were injected with primary tumor cells from patients [37].

The RGD peptide sequence recognizes the αvβ3 integrin molecule on tumor cell surfaces and has been widely investigated in various cancer models [42,43]. A cyclic peptide version called the cyclo(-RGDfv-) (cilengitide) was successfully shown in preclinical models to synergize with radiation and chemotherapy and resulted in increased tumor apoptosis and regression [44]. Data from phase 1/2 clinical trials of cilengitide in recurrent glioblastoma and newly-diagnosed glioblastoma have shown increased anti-tumor activity, either as a single agent or in concert with the chemo-drug temozolomide [45,46,47]. Another successful peptide-based targeting multifunctional NP system that has been translated into clinical trials is BIND-014, a prostate-specific membrane antigen (PSMA)-targeted polymeric NP that contains the chemotherapy drug docetaxel for the treatment of prostate cancer [48]. Preclinical studies showed that BIND-014 could deliver up to 10-times more docetaxel to tumor sites than non-specific drug delivery systems, correlating with tumor regression [48]. In the first clinical studies, data from patients suggested that BIND-014 had prolonged circulation and retention time in the vasculature and five-fold greater anti-tumor activity compared to clinically-administered docetaxel doses [49].

Aptamers are oligonucleotides with 15–40 bases that bind to various molecular targets, such as small molecules, proteins, nucleic acids and even cells, tissues and organisms [50]. AS1411 is an anti-nucleolin aptamer in phase 2 clinical development with a 26-base guanine-rich oligodeoxynucleotide with potential affinity to nucleolin, a nucleolar phosphoprotein that is overexpressed on the surface of various cancer cells [51,52]. Another aptamer that has been extensively used is the PSMA-specific A10 aptamer for engineering of targeted NPs in multiple prostate cancer preclinical and clinical studies. In all of these studies, targeted NPs complexed with chemotherapy drugs, such as docetaxel [53] or cisplatin [54], displayed higher tumor accumulation and more efficient tumor regression than non-specific NPs.

## 5. Cancer Diagnostics and Bio-Imaging

The intrinsic optical, thermal, electrical or magnetic properties of nanomaterials have been very efficacious in early stage detection and diagnosis of cancer. NP imaging probes can endow enhanced signal sensitivity, better spatial resolution and the capability to relay cellular and molecular information of the tumor mass.

Magnetic NPs have been used as the platform on which other functional molecules, such as fluorescent probes or radionuclides, can be attached to form dual modal imaging probes. Fluorescent dye-doped silica (DySiO_2_)-encapsulated magnetic NPs have been used to detect neuroblastoma cancer cells via MRI along with subcellular fluorescence imaging. Magnetic NPs have also been coupled to radionuclides, such as ^124^I, to engineer MRI-PET probes that can accurately detect lymph node metastasis in patients [55].

Hybrid modalities that have multiple imaging probes engineered into one NP structure synergistically combine the advantages offered by each specific imaging technique that can lead to earlier and more efficient detection of tumors and can also be used for real-time monitoring of therapeutic efficiency. A hybrid NP consisting of an iron oxide NP coated with a PEG-grafted chitosan polymer and conjugated to fluorescent Cy5.5 and a tumor targeting moiety was found to accumulate in medulloblastoma tumor tissue in transgenic mice. The signal intensity increased over the first 50 h after an intravenous injection and stayed steady for the next 70 h [56]. Similarly, a ^64^Cu radiolabeled iron oxide NP conjugated to the tumor binding RGD peptide was used for simultaneous MRI and positron emission tomography (PET) imaging of neuroblastoma tumors [57]. As discussed earlier, the RGD peptide recognizes the αvβ3 integrin that is expressed on the surface of tumor cells and involved in angiogenesis, an important step in tumor growth and metastasis. The dual probe NPs provided a greater signal-to-noise ratio and displayed higher tumor uptake and stronger visualization of the tumor.

Quantum dots have also been successfully employed in drug or gene delivery and imaging. A quantum dot-aptamer-doxorubicin (QD-Apt-DOX) conjugate was constructed by surface modification and attachment of the prostate tumor cell-specific A10RNA aptamer to the CdSe-ZnS core of the quantum dot that also was intercalated with doxorubicin. Upon internalization and release of doxorubicin, optical activation techniques can detect the fluorescence associated with this nanocomplex and can spatially locate the tumor, visualize drug delivery and uptake and detect the real-time response of the tumor cell to the drug [58].

Gold NPs have also been widely used in the fabrication of cancer-targeting multimodal drug delivery systems, because of the ease of synthesis, the presence of negative reactive groups on the surface that can be easily modified for conjugation and low toxicity [59]. A multifunctional NP system that consisted of gold NPs as the carrier, gemcitabine as the chemotherapy drug and the EGFR antibody (cetuximab) as a targeting moiety, was fabricated and resulted in significant inhibition of tumor growth in a pancreatic cancer model. Biodistribution of the NP delivery system was determined by inductively-coupled plasma analysis because of the presence of the gold, and it was found that targeted delivery of the NPs led to specific accumulation at tumor sites, resulting in the reduction of tumor growth [60].

The development of NPs that can serve as platforms for multicolor optical imaging provides the opportunity to obtain information about multiple molecular parameters associated with the cellular and metabolic processes of the tumor cell [61]. The use of imaging probes conjugated with biomarkers defining various physiological stages of tumor development can provide valuable information about the prognosis of the disease in a particular patient. This can be used to personalize their treatment regimen for maximum therapeutic efficacy.

## 6. Conclusions

Targeted and combinatorial NP therapies are therefore the future of cancer therapeutics and personalized medicine. Advances in genomics and proteomics have resulted in increasing information about the molecular profiles and biomarkers of various cancers. This information will aid in the fabrication of novel multifunctional NPs that can target tumor cells with more precision and specificity. Newer and more sophisticated multimodal imaging capabilities ensure early disease diagnosis, real-time monitoring and detection of its response to the treatment regimen. The power of nanotechnology in integrating all of these functionalities has caused a paradigm shift in the way cancer is treated and is inarguably the future of cancer therapy and cancer patient care.
